# TRAM-LAG1-CLN8 domain-containing protein TMEM56 regulates cell migration by changing intracellular ceramide levels

**DOI:** 10.1186/s12915-026-02614-7

**Published:** 2026-05-05

**Authors:** Benjamin Dorschner, Ralf Wiedemuth, Cornelia Richter, Marc Gentzel, Tobias Lindau, Gunes Ozhan, Gilbert Weidinger, Sebastian Brenner, Sebastian Thieme

**Affiliations:** 1 Department of Pediatrics, Faculty of Medicine and University Hospital Carl Gustav Carus, Technische Universität Dresden, Dresden, Germany; 2 German Center for Child and Adolescent Health (DZKJ), partner site Dresden/Leipzig, Dresden, Germany; 3 Division of Respiratory and Critical Care Medicine, University of Basel Children’s Hospital (UKBB), Basel, Switzerland; 4 Center for Regenerative Therapies, Technische Universität Dresden, Dresden, Germany; 5 Department of Molecular Biology and Genetics, İzmir Institute of Technology, Urla, İzmir, 35430 Türkiye; 6 İzmir Biomedicine and Genome Center (IBG), Dokuz Eylul University Health Campus, Inciralti-Balcova, İzmir, 35340 Türkiye; 7 Institute of Biochemistry and Molecular Biology, Ulm University, Ulm, Germany

**Keywords:** TMEM56, TLCD4, Ceramide synthase, Cell migration, Embryogenesis, Lipid metabolism, Hematopoiesis

## Abstract

**Background:**

Cell migration is a fundamental biological process essential for embryonic development and hematopoiesis. In a shRNA screen, we identified the TRAM-LAG1-CLN8 domain-containing transmembrane protein TMEM56 as a previously uncharacterized regulator of stromal cell-derived factor 1 (SDF-1)-mediated cell migration. This study investigates the molecular mechanisms underlying TMEM56 function.

**Results:**

TMEM56 is expressed in both murine embryonic and adult tissues, with enrichment in hematopoietic stem and erythroid progenitor cells. Lipidomic analysis reveals that TMEM56 modulates ceramide metabolism, particularly affecting levels of hexosylated ceramides. Co-immunoprecipitation assays indicate that TMEM56 physically interacts with ceramide synthase 2 (CerS2), suggesting a role in lipid signaling pathways.

**Conclusion:**

Our findings identify TMEM56 as a key regulator of cell migration, linking lipid metabolism with hematopoietic and developmental processes. These results provide novel insights into the molecular mechanisms governing migration.

**Supplementary Information:**

The online version contains supplementary material available at 10.1186/s12915-026-02614-7.

## Background

Cell migration is a fundamental biological process essential for embryonic development and hematopoiesis. In vertebrates, migration of hematopoietic stem and progenitor cells (HSPCs) may be regulated by stromal cell-derived factor 1 (SDF-1/CXCL12) binding to its receptor CXCR4, triggering signaling cascades that coordinate cytoskeletal rearrangements and membrane dynamics [1–4]. Increasing evidence indicates that lipid composition of the plasma membrane, in addition to chemokine gradients, critically influences CXCR4-dependent migration [5].

Sphingolipids represent a particularly important class of bioactive lipids that regulate cell motility. In particular, sphingosine-1-phosphate (S1P), glycosphingolipids, and sphingomyelin have been implicated in modulating SDF-1-dependent migration of HSPCs [6–15]. Central to this pathway, ceramide synthases (CerS) catalyze the formation of ceramides that serve as intermediates for both structural sphingolipids and signaling molecules including S1P [16, 17].

The catalytic center of CerS is the TRAM/LAG1/CLN8 (TLC) domain, which is also present in several other proteins, including CLN8, TLCD1, and TMEM56 [18–21]. While CLN8 and TLCD1 have been linked to lipid metabolic and regulatory activities [18, 22], the physiological role of TMEM56 remains unknown. TMEM56 is a highly conserved six-pass transmembrane protein (ENSGT01010000222313) with emerging associations to hematopoiesis, heme metabolism, and lipid biology [23–25], yet its mechanistic function has not been characterized.

In this study, we provide a comprehensive characterization of TMEM56 and its role in embryogenesis and hematopoiesis. Using a genome-wide shRNA screen, we identified TMEM56 as a novel regulator of cell migration towards SDF-1 and sphingolipid metabolism. Notably, our findings indicate that TMEM56 functionally interacts with CerS2, suggesting a mechanistic link between TMEM56 activity and altered lipid metabolism. We demonstrate that TMEM56 is expressed in long-term hematopoietic stem cells (LT-HSCs) and erythropoietic progenitor cells.

## Results

### TMEM56 regulates hematopoietic cell migration

**Fig. 1:**
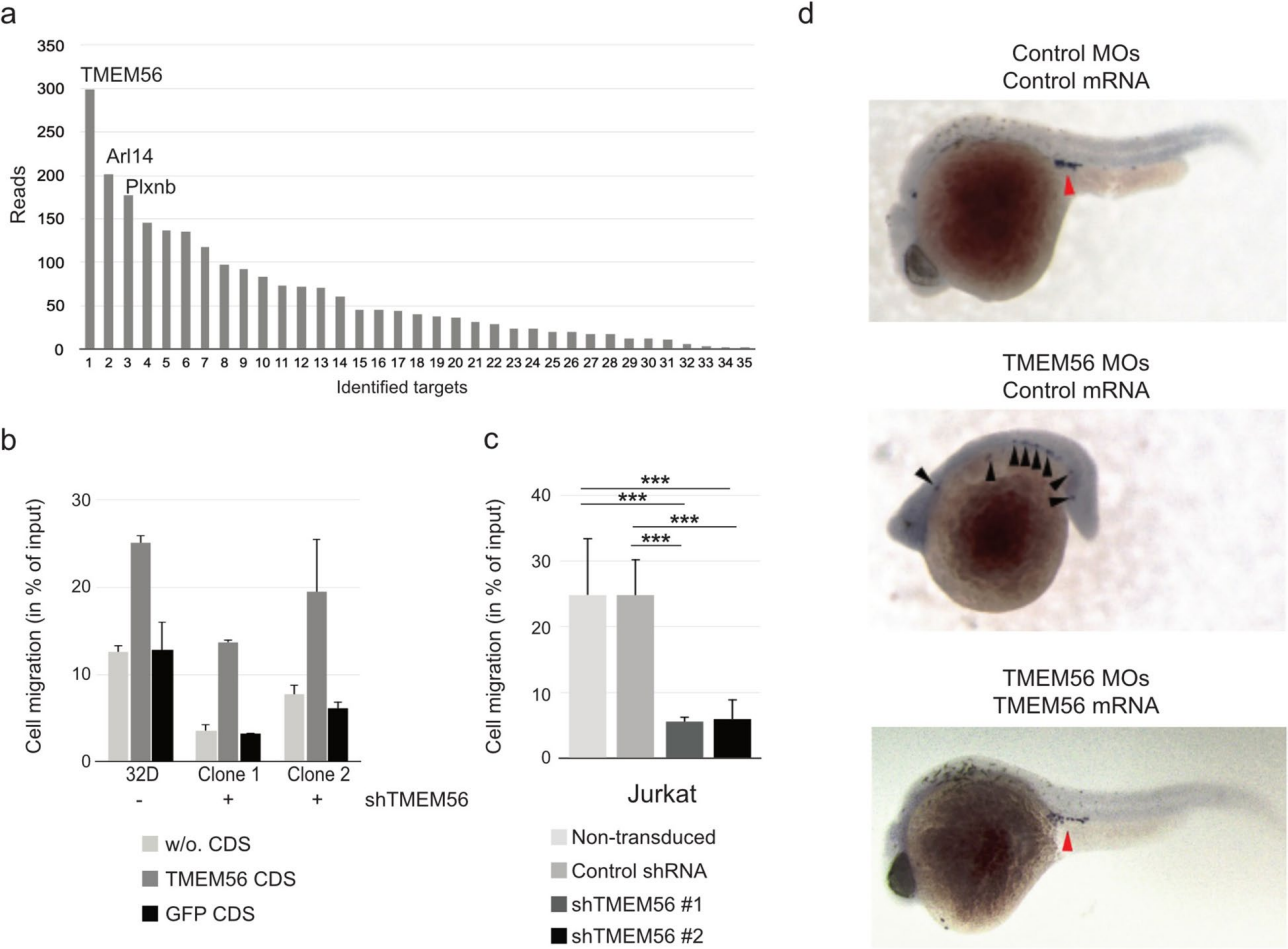
TMEM56 is critical for cell migration. **a** Distribution of migration relevant target genes identified in a genome-wide loss of function shRNA screening assay. **b** Migration capability of murine 32D cells (32D) and 32D cells expressing TMEM56 specific shRNA (clones 1 and 2). All cells were co-transduced with either TMEM56 CDS, GFP control CDS, or expressed no additional CDS (w/o). Mean values and standard deviation (SD) of three independent experiments are given. **c** Migration potential of TMEM56-depleted human Jurkat T cells. Jurkat cells were transduced with lentiviral vectors expressing either a control shRNA or TMEM56-specific shRNA. Data are presented as percentage values ± SD. Differences among groups were assessed by one-way ANOVA, followed by pairwise t-tests with Bonferroni correction to identify significant differences. Significance is indicated as: *p ≤ 0.05, **p ≤ 0.01, ***p ≤ 0.001, ****p ≤ 0.0001; ns = not significant.** d** Representative images of zebrafish embryos injected at the 1-cell stage with a pool of the three TMEM56-specific morpholinos (TMEM56 MOs) or co-injected with MO-resistant TMEM56 variant *tlcd4b* mRNA. The red arrowhead indicates the normal position (main cluster) of the primordial germ cells (PGC) in the embryo; black arrowheads show ectopic positions

To identify new regulators of SDF-1/CXCR4-dependent cell migration, we transduced murine myeloid progenitor 32D cells with the murine pRetroSuperCam shRNA library [26] and performed a transwell migration assay to screen for cells that had lost their migration capability. After 18 rounds of migration, DNA from non-migrated 32D cells was sequenced (see Additional file 1: Tab. S1). In 299 out of 2244 acquired reads, a TMEM56 targeting shRNA sequence was identified in the non-migrating cell population corresponding to a total of 13.3% of identified shRNA sequences (Fig. 1a). No TMEM56 shRNA-containing cell clone was detected among all shRNA library transduced single-cell clones of the cultured-only control population. To confirm the role of TMEM56, we transduced both murine 32D and human Jurkat cells with TMEM56 shRNA and control shRNA, respectively. TMEM56 shRNA containing 32D clones and Jurkat cells exhibited a reduced migration capability in transwell migration assays (Fig. 1b, c). The migration capability was rescued by overexpression of the TMEM56 coding sequence (CDS) but not the control GFP CDS (Fig. 1b).

### Primordial germ cell migration in zebrafish

Since TMEM56 is conserved among species (see Additional file 2: Fig. S1), we employed zebrafish as an exploratory in vivo system to interrogate SDF-1/CXCR4-dependent cell migration. Using antisense morpholino oligonucleotides (MOs), we assessed primordial germ cell (PGC) migration during early development according to [27] (see Additional file 3). Injection of pooled TMEM56-specific antisense MOs into 1-cell stage zebrafish embryos resulted in severe developmental abnormalities at 24 h post fertilization (hpf), including defects in body axis elongation and head formation. These phenotypes are suggestive of perturbed stem cell migration processes during gastrulation and somitogenesis (Fig. 1d).

### Identification of TMEM56-expressing cells and tissue in a mouse model

**Fig. 2:**
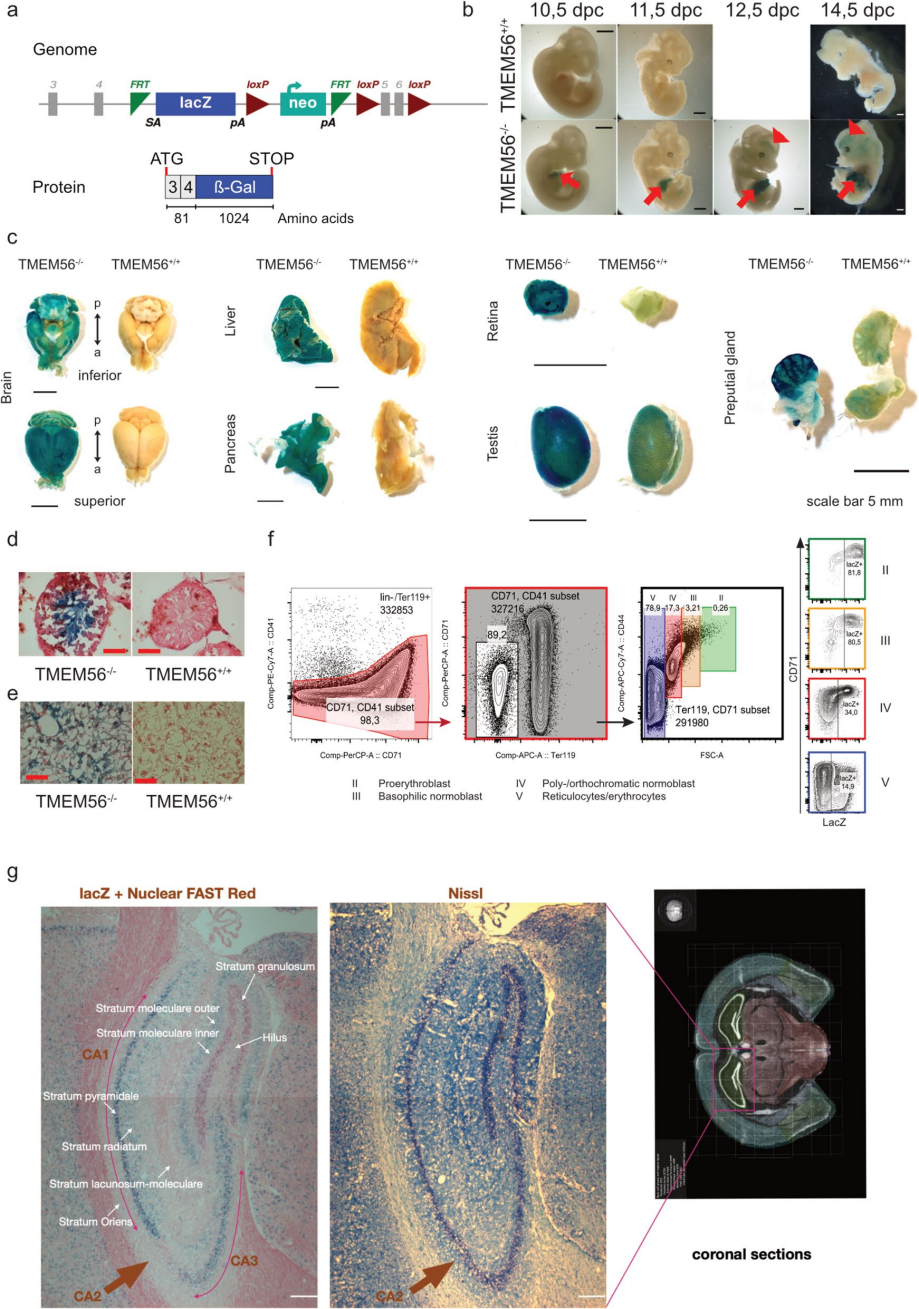
TMEM56 expression in embryonic and adult mouse tissue. **a** Schematic representation of the TMEM56 allele containing the lacZ reporter construct. The cassette induces a mutation in the TMEM56 mRNA, resulting in a null allele. Note that the incorporated β-Galactosidase (β-Gal) is linked to endogenous TMEM56 expression. **b** LacZ expression in the mouse embryo, observed in the fetal liver (arrow) and the developing brain (arrowhead). dpc—days post coitum. Scale bar: 1 mm. **c** Indirect analysis of TMEM56 expression in adult tissue of TMEM56^−/−^ and TMEM56^+/+^ mice using the tethered reporter gene (LacZ). Combined Nuclear Red/﻿β-Gal staining of transverse cryosections of adult testis (**d**) and adult liver (**e**) from TMEM56^−/−^ and TMEM56^+/+^ mice. Scale bar: 50 µm. **f** Analysis of TMEM56 expression during erythropoiesis. Representative image of a flow cytometry-mediated LacZ analysis in murine lin-/Ter119 + bone marrow cells derived from TMEM56^−/−^ mice. Gating strategy includes the removal of doublets and dead cells. **g** TMEM56/LacZ expression in the hippocampus (coronal section) of a TMEM56^−/−^ mouse. Cryosections were counterstained with Nuclear Fast Red. Scale bar: 200 μm

Given the migratory phenotype observed upon knockdown of *tmem56* paralogs in zebrafish, we next investigated TMEM56 expression and function in a mammalian model. We generated knock-out (KO) mice carrying a transcriptional stop cassette with an integrated *lacZ* reporter gene between FRT sites in intron 4 upstream of frameshifting exon 5 of TMEM56 flanked by loxP sites (Fig. 2a). This design produced a fusion transcript of exons 3–4 of Tmem56 and lacZ, followed by a polyadenylation signal, thereby terminating TMEM56 transcription. Unexpectedly, unlike the embryonically lethal phenotype and pronounced primordial germ cell migration defects observed in zebrafish morphants, Tmem56 knockout mice displayed no obvious early developmental or migratory abnormalities.

To confirm the functional disruption of the *Tmem56* allele, we assessed transcript and protein levels in adult tissues. qRT-PCR analysis using primer pairs covering exons 7 and 8 revealed residual *Tmem56* expression of approximately 20–25% of wild-type levels in liver samples of homozygous mutants (see Additional File 2: Fig. S2a). Western blot analysis using an antibody specific for the C-terminal region of TMEM56 confirmed the absence of full-length protein in homozygous mutants. No shorter protein variants corresponding to exons 7–8 were detected (see Additional File 2: Fig. S2b), indicating effective loss of TMEM56 protein. Together, these data demonstrate that the allele represents a strong hypomorphic, and likely null, mutation. β-galactosidase activity faithfully reflected endogenous Tmem56 promoter activity, but no overt phenotypic abnormalities were observed even in homozygous animals under standard laboratory conditions, suggesting that compensatory mechanisms or functional redundancy may mitigate the loss of TMEM56.

We next analyzed the spatial and temporal expression pattern of TMEM56 in embryonic (Fig. 2b) and adult (Fig. 2c) tissues. Homozygous TMEM56^−/−^ as well as heterozygous TMEM56^+/−^ mice and embryos were processed in parallel. LacZ-stained embryos exhibited specific expression in the fetal liver beginning at 10.5 days post fertilization (dpc), and in the craniofacial regions of the developing brain at 12.5 dpc (Fig. 2b). In adult tissue of five-month-old TMEM56^−/−^ mice, a strong LacZ expression was verified in the brain, liver, retina, testis, preputial gland and blood (Fig. 2c). The spatial and temporal patterns of β-galactosidase staining were indistinguishable between TMEM56^−/−^ and TMEM56^+/−^ tissue samples. However, the signal intensity in homozygotes was consistently higher. Therefore, figures show homozygous tissue unless stated otherwise.

No expression was detected in heart, kidney, lung, eye and thymus (data not shown). In cryosections of the testes, we were able to detect staining at the cellular level in the lumen of the seminiferous tubules and in the maturing spermatids (Fig. 2d). In liver sections, X-Gal signals could be detected in hepatic stellate cells (Fig. 2e).

Because TLC domain-containing protein CLN8 is expressed in the brain and is associated with neuronal ceroid lipofuscinosis [28], we investigated TMEM56 expression in the murine brain. Our analysis revealed strong X-Gal staining in the brain, particularly in the pyramidal layer of the hippocampus, the granular layer of the cerebellum and layers 1, 2, 3 and 6 of the isocortex, with specific staining of hippocampal regions CA1 and CA3 but not CA2 (Fig. 2g). Except for a reduced staining intensity, there were no differences between TMEM56^−/−^ and TMEM56^+/−^ animals. Nissl staining confirmed that hippocampal structures, including all three regions CA1-3, were intact in TMEM56^+/+^, TMEM56^+/−^, and TMEM56^−/−^ animals (data not shown).

Since we identified TMEM56 as a novel regulator of SDF-1/CXCR4-dependent cell migration, we analyzed its expression in adult hematopoiesis using a LacZ reporter system to determine which hematopoietic populations might be directly influenced by TMEM56. In lineage-depleted bone marrow (BM) cells from TMEM56^−/−^ and TMEM56^+/+^ mice, a specific expression of the *lacZ* reporter gene was detected in hematopoietic stem cells (HSCs), in a population of common lymphoid progenitor (CLPs) cells (data not shown), and in erythropoietic cells. In detail, using a CD105/CD150 and a CD34/CD135-mediated approach on LSK (lin-, Sca1 +, CD117 +) cells, LacZ expressing cells were found within the long-term HSC (LT-HSC) population (see Additional file 2: Fig. S3a, b). The CD105/CD150-mediated approach further enables hierarchical analysis of myeloid progenitor cells [29]. The earliest detectable LacZ expression in the myeloid lineage was observed in the erythroid colony-forming unit (CFU-E) population but not in granulocyte–macrophage progenitors (GMP) (see Additional file 2: Fig. S3b). Cellular breakdown of erythropoiesis revealed that 72.1% of proerythroblasts, 68.3% of basophilic normoblasts, 30% of poly-/orthochromatic normoblasts, and 11.3% of reticulocytes/erythrocytes expressed LacZ (Fig. 2f, see also Additional file 2: Fig. S3c). *LacZ* reporter gene expression allows for indirect estimation of TMEM56 expression levels. LacZ expression was at least doubled in proerythroblasts (18.6 ± 3.8 a.u.) and basophilic normoblasts (13.3 ± 4.2 a.u.) compared to poly-/orthochromatic normoblasts (6.3 ± 0.9 a.u.) and reticulocytes/erythrocytes (4.2 ± 0.8 a.u.) (Fig. 2f, see also Additional file 2: Fig. S3d). Of note, owing to the lack of DNA in reticulocytes/erythrocytes remaining LacZ activity might be based on deposited *lacZ* mRNA or LacZ protein.

In summary, TMEM56 is specifically expressed in erythropoietic progenitor and hematopoietic stem cells in the blood, as well as in the brain, retina, liver, and testis. Histological analyses of organs and tissues showing TMEM56 expression revealed no detectable morphological abnormalities, and the mouse model did not exhibit other phenotypic defects.

### Intracellular localization and characterization of TMEM56 using transgenic cell lines

**Fig. 3:**
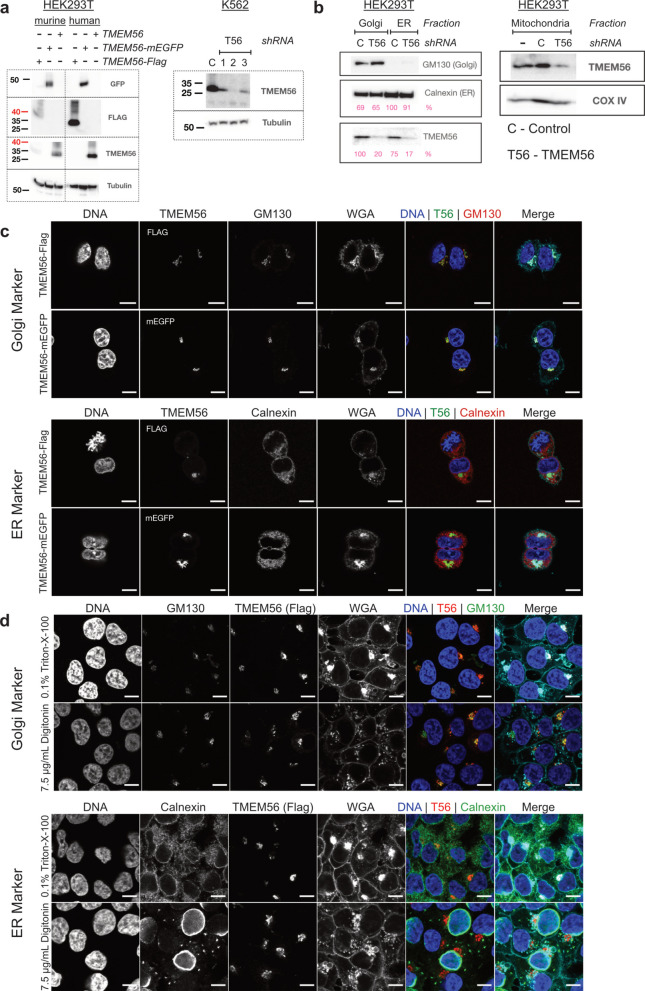
Intracellular localization of TMEM56. **a** Representative western blot analysis of TMEM56 expression in HEK293T and K562 cells. *Left panel:* HEK293T cells overexpressing native TMEM56 or FLAG or GFP-tagged TMEM56; anti-GFP and anti-FLAG used as positive controls. *Right panel:* endogenous TMEM56 expression is abolished by TMEM56-specific shRNA. All blots were cropped from the same original gel and probed separately with the indicated antibodies. Tubulin was used as loading control. **b** Detection of native TMEM56 by Western blot in isolated cell fractions. Markers for the endoplasmic reticulum (Calnexin), Golgi apparatus (GM130), and mitochondria (COX IV) are shown. **c** Cellular localization of TMEM56. Representative confocal immunofluorescence images of HEK293T cells constitutively expressing C-terminal Flag or GFP-tagged TMEM56. Cell compartments were stained using Hoechst for DNA, GM130 for Golgi, Calnexin for ER, and WGA for cell borders. TMEM56 was detected via the GFP signal or anti-Flag antibody. **d** Orientation of the TMEM56 protein in the Golgi membrane. The C-terminus points into the cytosol. A comparison was made between complete permeabilization of all membranes using 0.1% Triton X-100 and selective permeabilization of the cell membrane using digitonin. The epitope of the GM130 antibody is cytosolic, whereas the epitope of the Calnexin antibody is within the lumen of the ER. Scale bar: 10 μm

We generated a rabbit polyclonal antibody against a C-terminal peptide of TMEM56, capable of detecting both human and murine protein at endogenous levels (Fig. 3a, b). Target specificity was confirmed by mass spectrometry and by shRNA-mediated knockdown of TMEM56. The antibody recognizes native TMEM56 but does not detect C-terminally tagged fusion proteins, likely due to steric masking of the epitope (Fig. 3a).

To detect native TMEM56 protein in cellular compartments, we isolated ER and Golgi fractions via sucrose density gradient centrifugation and mitochondria through bead-mediated isolation. Strong TMEM56 bands were found in the Golgi fraction and a weaker signal in ER and mitochondrial fractions (Fig. 3b). We used GFP- and Flag-tagged TMEM56 variants for intracellular localization analysis. In line with our western blot results on subcellular fractions, TMEM56 localization in the Golgi apparatus was confirmed for both Flag- and GFP-tagged proteins (Fig. 3c). Selective permeabilization with digitonin [30] revealed TMEM56 stained together with cytosolic epitopes of GM130 but not luminal Calnexin (Fig. 3d), indicating that the C-terminus of TMEM56 protrudes into the cytosol.

### Analysis of TMEM56 altered cellular lipid profiles

**Fig. 4:**
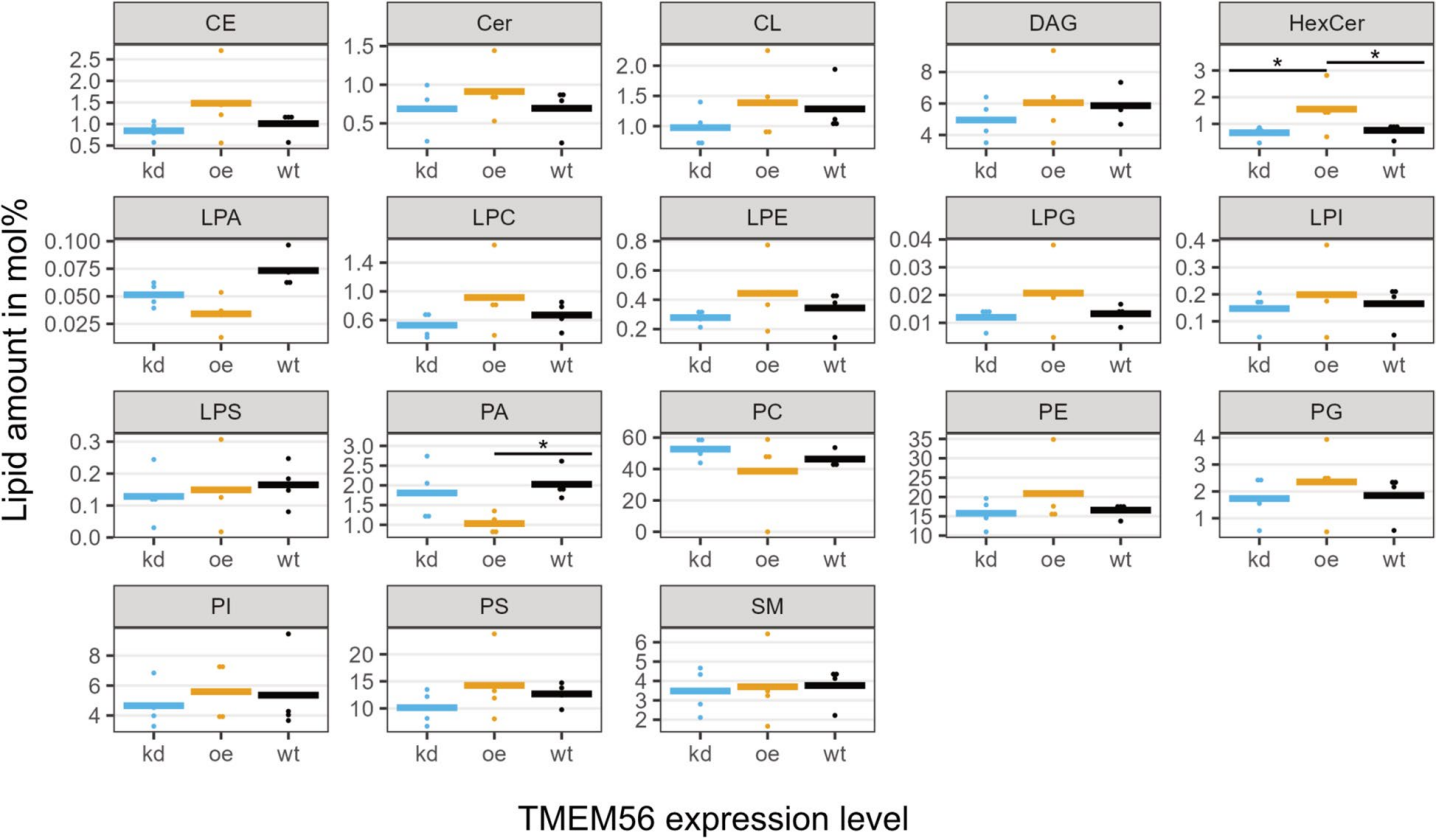
TMEM56 expression alters lipid profile. Dot plot showing lipid levels as a function of TMEM56 genotype (kd, knockdown; oe, overexpression; wt, wildtype; *n* = 4 per group). Thick lines indicate mean values; * indicates *p* < 0.05. Lipid abbreviations: CE, cholesteryl esters; Cer, ceramides; CL, cardiolipins; DAG, diacylglycerols; HexCer, hexosylated ceramides; LPA, lyso-phosphatidates; LPC, lyso-phosphatidylcholines; LPE, lyso-phosphatidylethanolamines; LPG, lyso-phosphatidylglycerols; LPI, lyso-phosphatidylinositols; LPS, lyso-phosphatidylserines; PA, phosphatidates; PC, phosphatidylcholines; PE, phosphatidylethanolamines; PG, phosphatidylglycerols; PI, phosphatidylinositols; PS, phosphatidylserines; SM, sphingomyelins

Based on the assumption that TMEM56 participates in lipid metabolism as other TLC domain-containing proteins do, we analyzed lipid profiles of HEK293T cells with different TMEM56 expression levels. Across all samples (*n* = 12 with 4 samples per genotype), 18 lipid classes were identified, independent of TMEM56 expression level. Among these, significant genotype-dependent differences were observed for hexosylceramide (HexCer) and phosphatidic acid (PA). HexCer species were increased in TMEM56-overexpressing cells (1.551 ± 0.950 mol%) compared to both wild-type cells (0.761 ± 0.268 mol%, *p* = 0.043) and knockdown cells (0.670 ± 0.260 mol%, *p* = 0.026), corresponding to mean fold changes of 2.04 (95% CI [1.07, 3.88]) and 2.32 (95% CI [1.18, 4.46]), respectively (Fig. 4). Notably, C20-HexCer levels showed a clear association with TMEM56 expression, being lowest in knockdown cells (0.0021 ± 0.0041 mol%) and highest in overexpressing cells (0.0187 ± 0.0096 mol%) compared to wild-type (0.0056 ± 0.0074 mol%, *p* = 0.027). In contrast, PA levels were significantly reduced in TMEM56-overexpressing cells (1.03 ± 0.26 mol% vs. 2.03 ± 0.41 mol% in wild-type, *p* = 0.017), with a mean fold change of 0.46 (95% CI [0.26, 0.69]). PA abundance in overexpressing cells did not differ significantly from knockdown cells, nor did knockdown levels differ from wildtype. No other lipid classes showed statistically significant changes.

### Identification of TMEM56 interacting proteins

**Fig. 5:**
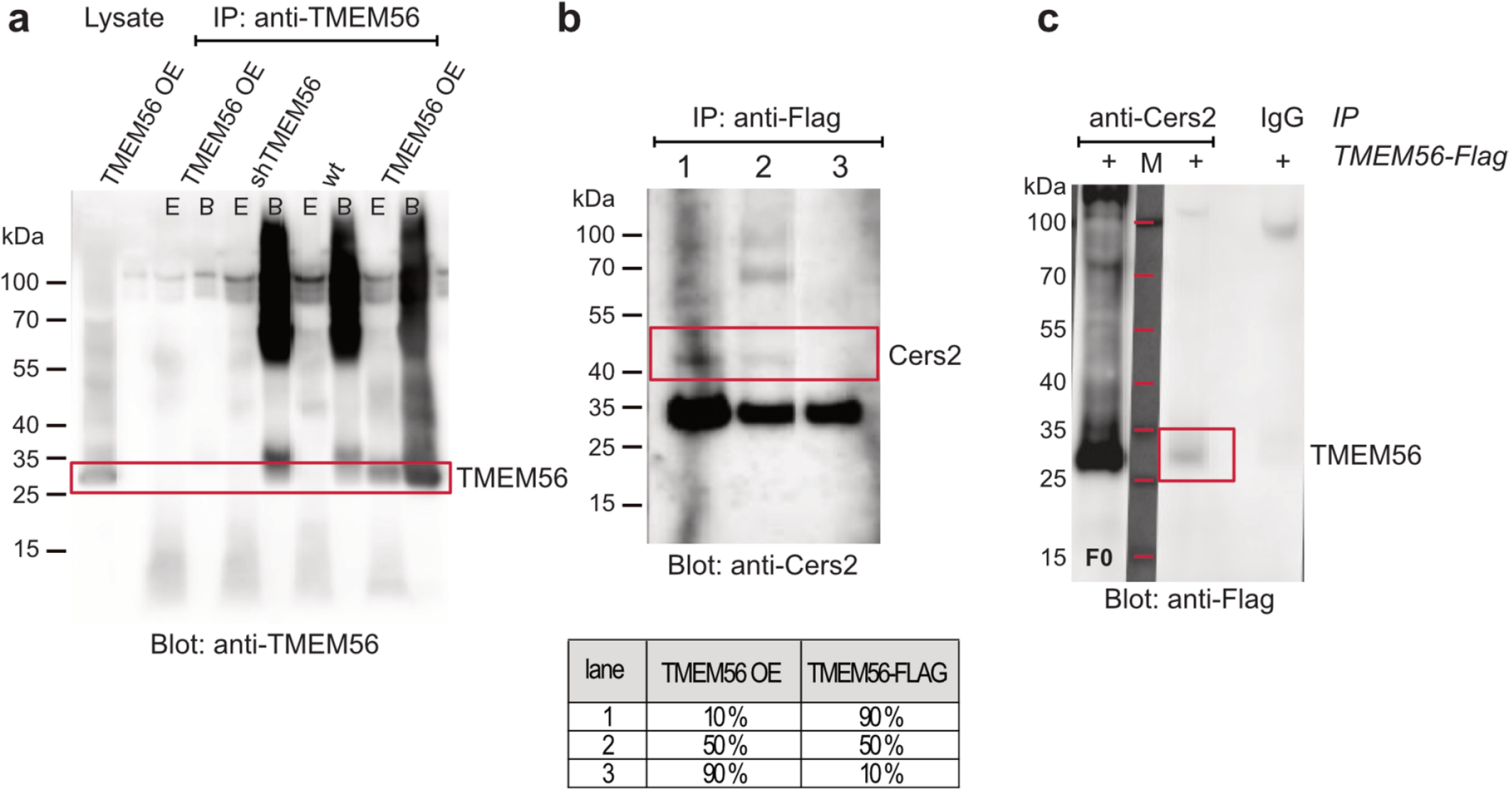
Co-immunoprecipitation of TMEM56 and CerS2 in HEK293T cells. **a** Representative Western blot of an immunoprecipitation (IP) of TMEM56 using our custom TMEM56 antibody. Lysates from HEK293T cells overexpressing TMEM56 (OE) or with TMEM56 knocked down by shRNA were used as input for the IP. The same antibody was used for the detection of TMEM56 protein in the Western blot as for the IP. "E" denotes the eluate obtained through acidic buffer, and "B" denotes the bead lysate. **b** Co-immunoprecipitation of TMEM56-Flag using an anti-Flag antibody, followed by the detection of CerS2 by Western blot. Different ratios of HEK293T-TMEM56 and HEK293T-TMEM56-Flag lysates were used as input. **c** Reverse co-immunoprecipitation of CerS2 from HEK293T-TMEM56-Flag lysates with subsequent detection of TMEM56 using a Flag antibody. "F0" represents the eluate from the first wash step, and “M” represents the protein marker

Increased levels of hexosylated ceramide species and the fact that the TLC domain of TMEM56 is shared by other proteins, especially ceramide synthases, led us to investigate possible interactions with other TLC domain-containing proteins. First, we used whole cell extracts from HEK293T cells with varying levels of TMEM56 expression. Although our antibody specifically detected and successfully precipitated TMEM56, detection conditions prevented the co-precipitation of potential interacting proteins (Fig. 5a). Therefore, we performed co-immunoprecipitation experiments on whole cell extracts from HEK293T cells expressing Flag-tagged TMEM56. Co-IP eluates were used for mass spectrometry to identify interacting proteins. TMEM56 peptide sequences were highly enriched in TMEM56-Flag expressing eluates compared to control samples (log2 fold changes 11.0). In total, 150 different proteins were identified as potential interactors with a log2 fold enrichment > 2 compared to control samples (data not shown). Among others, the presence of ceramide synthase 2 (CerS2), very-long-chain (3R)−3-hydroxyacyl-CoA dehydratase 3 (HACD3), and ADP-forming succinate CoA ligase suggested a role for TMEM56 in lipid metabolism. The association with CerS2 was further supported by western blot detection in Flag-TMEM56 Co-IP eluates from competition experiments with varying tag-to-untagged TMEM56 ratios (1:9, 1:1, 9:1; Fig. 5b). The interaction was confirmed when TMEM56 was detected in eluates of reverse CoIPs against CerS2 (Fig. 5c).

## Discussion

Through a shRNA screen [31], we identified TMEM56 as a regulator of hematopoietic stem cell (HSC) migration. We have shown that TMEM56 is abundantly expressed in murine HSCs, particularly long-term HSCs and early erythroid progenitors, consistent with prior reports [23]. TMEM56 expression appears to be regulated by key erythropoietic factors such as Runx1, Klf1, and GATA1/2 [32–37]. Regarding subcellular localization, we found TMEM56 predominantly expressed in the Golgi apparatus, a known ceramide glycosylation site [38], with lower levels in the ER and mitochondrial sites.

We employed two in vivo models to explore TMEM56 functionality. In zebrafish, morpholino-mediated knockdown was associated with impaired SDF-1/CXCR4-dependent PGC migration during embryonic development. In contrast, constitutive knockout mice did not display overt early developmental abnormalities. While these findings point to context-dependent effects, both models are consistent with a potential role for TMEM56 in SDF-1–directed stem cell migration. In zebrafish, knockdown was accompanied by altered PGC localization, whereas in a murine HSC model, loss of TMEM56 reduced SDF-1–directed migration. This migratory phenotype correlated with changes in lipid composition: TMEM56 overexpression increased levels of hexosylated ceramides (HexCer), particularly C20-HexCer species, whereas knockdown reduced them. Together with the reported heteromerization of TMEM56 with CerS2, these data are compatible with TMEM56 acting as a modulator of CerS2 activity. This interpretation aligns with the concept that the first glycosylation step of ceramide represents a rate-limiting event in glycosphingolipid metabolism [39, 40] and with evidence that TLC domain-containing proteins form heteromeric complexes influencing enzyme activity [41, 42].

Ceramide metabolism has been implicated in regulating migratory behavior, although its effects appear highly context-dependent. For instance, CerS2 suppresses migration in tumor and Schwann cells, yet promotes neutrophil migration [43–45]. By contrast, CerS6 knockdown reduces migration in lung and epithelial cancer cells, whereas its overexpression enhances migration in breast cancer cells [46]. Importantly, ceramide glycosylation also acts as a critical regulator: ECHS1-mediated glycosylation promotes colorectal cancer cell migration, and loss of ECHS1 abolishes this phenotype [47]. In the specific context of CXCR4 signaling, glycosylated ceramides stimulate migration via the SDF-1–CXCR4 axis, while inhibition or knockdown of glycosylceramide synthase blocks migration [8, 14]. Functionally, this aligns with our finding that TMEM56 knockdown reduces C20-HexCer, although the precise molecular link remains unresolved. One possibility is that TMEM56-dependent lipid remodeling alters lipid raft composition, affecting CXCR4 anchorage and signaling which depends on lipid raft sphingolipid composition [6, 9, 48, 49]. Experimental validation of this hypothesis, for example through lipid raft analyses, through inhibition of glucosylceramide synthases or the use of catalytically inactive TMEM56 mutants, remains an important future direction.

## Limitations

The zebrafish experiments presented in this study were performed using translation-blocking morpholino oligonucleotides together with control morpholinos, dose–response assessment, and mRNA rescue experiments. While these approaches support the specificity of the observed phenotypes, additional validation strategies, such as the use of multiple independent morpholinos per gene or CRISPR/Cas9-generated mutants, were not included. Thus, the zebrafish findings support an in vivo role of TMEM56 in cell migration and suggest a potential role during embryonic development, while warranting further investigation.

## Conclusion

Our study identified TMEM56 as a critical regulator of sphingolipid metabolism and hematopoietic stem cell migration. TMEM56 modulates hexosylceramide levels, likely through interaction with CerS2, uncovering a mechanistic link between TMEM56, ceramide metabolism, and CXCR4-mediated migration.

## Methods

### Cell lines & culture

Murine 32D cells (ATCC CRL-11346, non-authenticated, mycoplasma negative) were cultured in RPMI 1640 medium (PAA Laboratories, Pasching, Austria) supplemented with murine recombinant interleukin (IL)−3 (100 ng mL^−1^; R&D Systems, Minneapolis, USA). Human Jurkat T cells (ATCC TIB-152, non-authenticated, mycoplasma negative) were cultured in RPMI 1640 medium. Human embryonic kidney (HEK) 293 T cells (ATCC CRL-321, non-authenticated, mycoplasma negative) were cultured in IMDM medium (Biochrom, Berlin, Germany). All media were supplemented with 10 (v/v) % fetal calf serum (HyClone, South Logan, UT), 2 mM l-glutamine, 100 U mL^−1^ penicillin and 100 µg mL^−1^ streptomycin (PAA Laboratories). Cells were cultured at 37 °C in a humidified atmosphere with 5 (v/v) % CO2.

### Large-scale shRNA library screen

To generate a library containing virus-vector particles, 293 T cells were transfected using polyethylenimine with the murine pRetroSuperCam shRNA library targeting approximately 15 000 murine genes as described [26]. pRetroSuperCam vector without shRNA insert was used as control. Transduction of 32D cells was performed as described elsewhere [50].

### Transwell migration assay and identification of relevant genes

96-well migration assays were carried out using the ChemoTx system (pore size: 5 μm; Neuro Probe, Gaithersburg, USA) as described previously [31]. SDF-1 (100 ng mL^−1^) was added to the lower chamber. Cells (3 × 10^4^) were placed on the top of the membrane. Migration was stopped after 90 min. Migrated cells were collected from the bottom chamber and counted using flow cytometry. Genomic DNA from non-migrated 32D cells was isolated using DNeasy Blood & Tissue Kit (Qiagen) according to the manufacturer’s instructions. ShRNA-containing PCR fragments were amplified with PAN Hot Start DNA Polymerase (PAN-Biotech; primers: pReSu_amp_h, pReSu_amp_r; see Additional file 1: Tab. S3). PCR products were purified with QIAquick PCR purification Kit (Qiagen) and used for subsequent nested-PCR (primers: pReSu_h, pReSu_r). Nested PCR products were used for next generation sequencing using a GS-Flx 454 system (Inqaba Biotechnical Industries, Hatfield, South Africa).

### Generation of lentiviral vectors containing TMEM56 CDS or shRNA

pLKO.1, Lv241, Lv105, Lv193 and LVRH1MH vectors encoding shRNA targeting human or murine TMEM56 were obtained from OpenBiosystems (GE Healthcare Europe, Freiburg, Germany), Addgene (Watertown, USA) or GeneCopoeia (Rockville, USA) respectively (see Additional file 1: Tab. S4). Murine TMEM56 CDS was amplified from a cDNA library. An IRES-*GFP* cassette was added to confer GFP reporter expression and the construct was cloned into the lentiviral transfer vector pRRL.SIN.cPPT.SFFV.GFP.WPRE [51]. An empty vector served as control. Correct sequences of all vector plasmids were verified by nucleotide sequencing. To produce lentiviral-vector particles, HEK293T cells were transfected with a lentiviral transfer vector in combination with the packaging plasmids psPAX2 and pVSVg using PEI [52]. Lentiviral particle containing medium was collected 48 h after transfection. Target cells were infected with lentiviral vector particles (0.5 × viral supernatant) overnight in the presence of 1 µg mL^−1^ protamine sulfate (Merck, Darmstadt, Germany).

### Generation of cell lines stably expressing shRNA against human TMEM56

HEK293T, Jurkat and K562 cells stably expressing shRNA against human TMEM56 as well as the respective controls were generated as follows. 1 × 10^5^ cells were incubated overnight with the concentrated virus supernatant in a total volume of 1.5 ml of the usual cell culture medium containing 1 µg mL^−1^ protamine sulfate. Vectors containing shRNA or the human TMEM56 CDS were used at equal titers as their respective controls (see Additional file 1: Tab. S4 for a detailed list of the plasmids). Virus supernatant was removed by pelleting and resuspending the cells in medium. Two days after transduction, puromycin or hygromycin was added to transduced cells at a concentration of 2 µg mL^−1^or 100 µg mL^−1^, respectively.

### Primordial germ cell migration in zebrafish

Three translation-blocking morpholino oligonucleotides (MOs) targeting zebrafish orthologs of human *TMEM56* were synthesized by Gene Tools (Philomath, USA). Specifically, the MOs targeted *tlcd4b* (formerly *tmem56/tmem56b*), *tlcd4a* (formerly *tmem56a*), and *transmembrane protein 56-B-like isoform X1*. Standard control oligo (also from Gene Tools, Philomath, USA) was used as negative control MO. Capped, wobble-mutated *tlcd4b* mRNA and *egfp* mRNA (as control) were synthesized in vitro using the mMESSAGE mMACHINE SP6 Kit (Thermo Fisher Scientific, Dreieich, Germany) and used for rescue experiments. MOs (15 ng each) and *tlcd4b* mRNA (150 pg) were co-injected into the cytoplasm of zebrafish embryos at the 1-cell stage. *Vasa* mRNA in situ hybridization of zebrafish embryos to detect PGCs was performed as described previously [53]. MOs sequences are listed in Additional file 1: Tab. S3.

### Generation of TMEM56-knockout mice

Tmem56 knockout mice were generated using murine embryonic stem cells containing a “knockout-first” allele (C57BL/6N background) obtained from the International Mouse Phenotyping Consortium (IMPC) [54, 55]. The targeted allele (designated Tmem56 < tm1a(EUCOMM)Wtsi >) contains a gene-trap cassette inserted into intron 4 of the Tmem56 locus. This cassette includes a splice acceptor (SA), a lacZ reporter gene, and a polyadenylation signal (pA), flanked by FRT sites, and followed by a floxed exon 5. The SA–lacZ–pA cassette interrupts normal splicing, leading to the inclusion of the reporter and premature transcriptional termination. As a result, exons downstream of the insertion site are not transcribed, producing a truncated fusion transcript and functionally inactivating the Tmem56 gene. This configuration allows for both reporter-based expression analysis and gene disruption at the mRNA level, resulting in a null allele. Clones were injected into C57Bl/6 J blastocysts at the transgenic core facility of the Max Planck Institute of Molecular Cell Biology and Genetics. Male chimeras were crossed to C57Bl/6 J females and progeny were genotyped for germline transmission. For time-specific embryo preparation, a positive plug-check on the morning after mating was counted as day E0.5. Preparations were done at the indicated stages. Experiments were performed with permission from the local authorities. Genotyping was performed with isolated genomic DNA from embryonic and adult tails, blood and other tissues. Genomic DNA from all tissues excluding blood was isolated as described previously [31]. Genomic DNA from blood was isolated with the DNeasy Blood&Tissue kit (Qiagen, Hilden, Germany) according to the manufacturer’s instructions. PCR fragments were generated with GoTaq (TMEM56; Promega, Madison, USA) or DreamTaq (gender; Thermo Fisher Scientific) DNA polymerase. Applied primer sequences are listed in Additional file 1: Tab. S3. PCR fragments were analyzed by agarose gel electrophoresis.

### Preparation of murine bone marrow cells (BM)

To collect BM, mice were sacrificed by cervical dislocation. BM was collected by flushing femurs and tibia with 1 × PBS (0.5 mM EDTA, 1 (w/v) % bovine serum albumin (BSA)). Resuspended cells were filtered over a 70 µm nylon mesh to get a single cell suspension.

### Flow cytometry

Flow cytometric analyses were conducted using an LSR II flow cytometer (BD Biosciences, Heidelberg, Germany). Data were evaluated using FlowJo software (Version v10; FlowJo, Ashland, USA) including doublet discrimination and dead cell/debris exclusion. All antibodies against mouse cells were used at appropriate dilutions, as determined by previous titration. Dead cells were excluded by 4,6 diamidino-2-phenylindole (DAPI) staining (Sigma-Aldrich, Munich, Germany). Mouse blood cells were characterized by using the antibody panels described in Additional file 4. For an accompanied LacZ staining in flow cytometry, antibody-stained cells were labelled with the FluoReporter™ *lacZ* Flow Cytometry Kit (Thermo Fisher Scientific) according to the manufacturer’s instructions. FACS sorting of erythropoietic progenitor cells was carried out by using the erythropoiesis antibody panel (see Additional file 4) and a FACS Aria II SORP (BD Biosciences, Heidelberg, Germany).

### Indirect immunofluorescence analysis

Cells grown on poly-L-lysine (Merck, Darmstadt, Germany) coated glass slides were washed three times with HBSS containing Mg/Ca (Thermo Fisher Scientific) and fixed for 20 min with 4% (w/v) PFA in HBSS with Mg/Ca (Thermo Fisher Scientific) at 37 °C. The cells were then washed three times for 5 min with HBSS and stained with WGA-Texas Red (wheat germ agglutinin; Thermo Fisher Scientific) in HBSS (5 μg mL^−1^) for 10 min at 37 °C, followed by three additional washing steps with HBSS. Subsequently, the cells were permeabilized with 0.1% (v/v) Triton-X-100 in PBS for 15 min and blocked with 1% (v/v) BSA in PBS for 1 h at RT. For selective permeabilization of the plasma membrane, cells were incubated with 7.5 µg mL^−1^ Digitonin (Merck) in KMEH buffer (0.3 M sucrose, 0.1 M KCl, 2.5 mM MgCl_2_, 1 mM EDTA, and 10 mM Hepes, pH 6.9) for 15 min at RT, followed by a blocking step in 1% (v/v) BSA in PBS for 1 h at RT [21].

After blocking, cells were incubated with a primary antibody solution (1% (v/v) BSA in PBS) overnight at 4 °C, containing antibodies described in Additional file 4. After three 5-min washes with PBS, cells were incubated with a secondary antibody solution (see Additional file 4) for 1 h, followed by extensive washing cycles with HBSS. DNA was counterstained using HBSS containing Hoechst 33,342 (1 μg mL^−1^, Thermo Fisher Scientific).

Finally, the cells were washed twice with HBSS and once with double-distilled water before examination by confocal laser scanning microscopy. Coverslips were carefully removed and mounted with VECTASHIELD Antifade Mounting Medium (VectorLabs, Newark, USA) on glass slides. Microscopy was performed as described by Wiedemuth et al. [56]. Stained cells were imaged using a Leica SP5 inverse microscope (Leica, Wetzlar, Germany). Confocal images were collected at 405, 488, 543, and 594 nm with a 63 × NA1.4 objective lens. Image acquisition, shutter, Z-axis position, laser lines, and the confocal system were all controlled by Leica LAS AF software. Equivalent exposure conditions were used between samples. Depending on cell density, a digital zoom was applied to present numerous cells per image. Images were analyzed using Fiji software, with image processing including a median filter with a radius of 1 pixel to subtract background, and equal brightness and contrast adjustments between the samples [57].

### Histological analyses

For a whole-mount and adult tissue LacZ staining, explanted organs and embryos from sacrificed mice were washed in ice cold 1 × PBS and fixed in fresh 4 (w/v) % paraformaldehyde in 1 × PBS for 60 min at 4 °C and an additional 30 min at room temperature. Before fixation, tail tips from embryos were taken for genotyping. Fixed embryos and organs were washed in 1 × PBS and incubated at 37 °C for 3 h up to overnight in X-gal (1 mg mL^−1^ Carl Roth, Karlsruhe, Germany) staining solution including 2 mM magnesium chloride, 0.02% (w/v) NP40, 0.01% (w/v) sodium deoxycholate, 5 mM potassium ferrocyanide and 5 mM potassium ferricyanide. After staining, tissues were rinsed in 1 × PBS and post-fixed in 4% (w/v) paraformaldehyde. The intensity of the LacZ expression was evaluated as a function of the temporal appearance of the staining.

### Tissue preparation and staining procedures

Mouse brain, liver, and testis samples were embedded in OCT tissue-freezing medium (Leica) following equilibration in 15% and 30% (w/v) sucrose solutions. Cryosections of 10 µm thickness were prepared in the Histology and Electron Microscopy facility (CRTD, Dresden, Germany) using a Cryostat NX70 (Epredia, Dreieich, Germany). Adjacent sections from brain samples were subjected to either lacZ or Nissl staining, while liver and testis samples were stained exclusively for lacZ.

### Nissl staining

Cryosections were air-dried and stained for 2 min in 1% (w/v) toluidine blue (Thermo Fisher Scientific) dissolved in 0.5% (v/v) borax (Acros Organics, Geel, Belgium). Sections were rinsed briefly under running distilled water and differentiated through two changes of 96% ethanol for 2 min each, followed by quick immersion in 100% ethanol. Sections were cleared in two changes of xylene for 2 min each and mounted with coverslips.

### lacZ staining

Cryosections were fixed in 0.2% (v/v) glutaraldehyde (Carl Roth, Karlsruhe, Germany) at 4 °C for 10 min, then washed three times in lacZ wash buffer for 5 min each at room temperature. The lacZ wash buffer consisted of 0.1 M phosphate buffer (pH 7.4) supplemented with 2 mM MgCl₂, 0.02% (v/v) NP-40, and 0.01% (w/v) sodium deoxycholate. Staining was performed using a lacZ staining solution prepared by adding 5 mM potassium ferricyanide, 5 mM potassium ferrocyanide, and 1 mg/ml X-Gal to the lacZ wash buffer. Sections were incubated in the staining solution at 37 °C in a humidified chamber protected from light for 4–5 h.

Following staining, sections were washed three times in PBS for 5 min each, fixed in 4% (w/v) paraformaldehyde (Carl Roth) for 20 min at room temperature, and rinsed in PBS. Slides were washed twice in distilled water for 5 min and counterstained with Nuclear Fast Red (Carl Roth, Karlsruhe, Germany) for 5–10 min. After rinsing in tap water until clear and washing in distilled water for 2 min, slides were dehydrated through a graded ethanol series (50%, 75%, 90%, and 100%, 2 min each). Finally, slides were mounted with VectaMount (Vector Laboratories) and coverslips and stored at room temperature.

Slides were subsequently scanned using a Zeiss Axioscan.Z1 digital slide scanner.

### Subcellular fractionation of HEK293T cells

Golgi and endoplasmic reticulum (ER) fractions were isolated from cultured HEK293T cells using a sucrose density gradient under cold conditions to preserve organelle integrity. Cells were harvested by centrifugation (300 × g, 10 min, 4 °C) and washed sequentially in ice-cold phosphate-buffered saline (PBS) and a homogenization medium (HM) containing 0.25 M sucrose and 10 mM Tris⋅Cl, pH 7.4.

The cell pellets were resuspended in HM and gently homogenized with a tight-fitting Dounce homogenizer to minimize damage to intracellular organelles. Following homogenization, the lysate was adjusted to a final sucrose concentration of 1.4 M by adding a 2.0 M sucrose solution. The sample was carefully layered over a discontinuous sucrose gradient consisting of 1.2 M and 0.8 M sucrose layers, with an underlay of 1.6 M sucrose.

Centrifugation was performed at 110,000 × g for 2 h in a precooled ultracentrifuge. The Golgi fraction was recovered from the interface between the 0.8 M and 1.2 M sucrose layers, while the ER fraction was collected from within the 1.4 M sucrose layer. The collected fractions were diluted with HM, re-centrifuged, and the resulting pellets resuspended in HM.

Protein concentrations of the final suspensions were determined, and the fractions were aliquoted to a concentration of 1–5 mg protein mL^−1^ for subsequent analyses.

### Generation of TMEM56 antibody

The polyclonal anti-TMEM56 antibody was generated by immunizing rabbits with a synthetic peptide corresponding to a C-terminal region of the human TMEM56 protein (Eurogentec). As the antibody targets the native C-terminus, it does not detect TMEM56 constructs with C-terminal tags such as GFP or FLAG.

Rabbits of the breed white giants were initially immunized with emulsion generated with Freund’s complete adjuvant and TMEM56-specific peptide CQEKAKDSLQNGKLD coupled to KLH (GenScript, Rijswijk, Netherlands). Immunizations are repeated every three months with emulsions made with Freund’s adjuvant peptide. Serum was isolated from peripheral blood draws by centrifugation.

Anti-TMEM56 antibodies were affinity-purified at the Antibody Facility of the MPI-CPG, Dresden, from rabbit serum using the peptide CQEKAKDSLQNGKLD (GenScript) coupled to SulfoLink Coupling Gel (Thermo Fisher Scientific). The peptide was dissolved in 50 mM Tris, 5 mM NaEDTA, pH 8.5, and conjugated to the coupling gel according to the manufacturer’s instructions. Non-specific binding sites were blocked with 50 mM cysteine in 50 mM Tris, 5 mM NaEDTA, pH 8.5. Serum was diluted 1:1 with 20 mM Tris, pH 7.5, and filtered through a 0.22 µm Millex-GV filter. Serum was passed over the peptide-conjugated column twice at a flow rate of 1 mL min^−1^ to enhance binding. After washing the column with PBS and high-salt wash buffer (PBS, 0.5 M NaCl, 0.1% (v/v) TritonX-100), bound antibodies were eluted with 0.1 M glycine, pH 2.6. Fractions were neutralized immediately with 2 M Tris–HCl, pH 8.5.

Antibody-containing fractions (A280 measurement) were pooled and concentrated using Amicon Ultra-30 K centrifugal filters, with buffer exchange to PBS, pH 7.8. Final antibody concentrations were measured via Nanodrop spectrophotometry.

### Cell lysis and western blotting

Cell lysis and Western blot analysis were performed as described elsewhere [58]. Briefly, for protein extraction, 10–100 µL of RIPA lysis buffer (150 mM sodium chloride, 1% (v/v) NP40, 0.5% (w/v) sodium deoxycholate, 0.1% (w/v) sodium dodecyl sulfate, 50 mM Tris HCL pH8) supplemented with protease inhibitors (1 µg mL^−1^ Leupeptin and 10 µg mL^−1^ Aprotinin) was added per 1 × 10⁶ cells. Samples were incubated on ice for 15 min, followed by sonication using a Hielscher UP200S ultrasonic processor (50% amplitude) with three 2-s pulses, allowing at least 1 min on ice between each pulse. Lysates were further incubated on ice for 15 min before centrifugation at 13,000 × g for 5 min at 4 °C. The supernatant was collected and stored at −20 °C. Protein concentration was determined using the Pierce 660 nm Protein Assay Kit (Thermo Fisher Scientific) according to manufacturer’s instructions.

Samples were denatured in NuPage Bolt LDS buffer and Sample Reducing Agent (Thermo Fisher Scientific) for 30 min at room temperature. Whole-cell lysates and subcellular fractions were loaded onto NuPage Bolt SDS polyacrylamide gels (Thermo Fisher Scientific), separated by gel electrophoresis, and transferred onto PVDF membranes (Merck, Darmstadt, Germany).

Membranes were washed three times for 10 min in 1 × TBS containing 2% (v/v) Triton X-100 and 0.5% (v/v) Tween 20 (TBS-TT) and blocked with 5% (w/v) milk powder or BSA in TBS-TT. Primary antibody incubation was performed overnight at 4 °C in blocking solution, using the antibodies listed in Additional file 4.

After primary antibody incubation, membranes were washed three times with TBS-TT and incubated for 1 h at room temperature with HRP-conjugated secondary antibodies (see Additional file 4). Following additional washes, signals were detected using Luminata Forte chemiluminescent substrate (Merck) and imaged with an Azure c300 system (Azure Biosystems, Dublin, USA). Unprocessed images with illumination times series and corresponding marker images are provided in Additional File 5.

### Co-Immune precipitation

Co-immune precipitation was done as described previously [59]. Briefly, all steps were performed on ice or at 4 °C unless stated otherwise. 1.5 mg Magnetic protein G Dynabeads (Thermo Fisher Scientific) were resuspended in PBS, washed three times with PBS using a static magnet, and then incubated with antibodies for coupling. Beads were incubated with 10 μg anti-TMEM56, 10 μg anti-FLAG (F1804, Merck) and 4 µg anti-CerS2 (Bethyl Laboratories, A303-193A, Montgomery, USA) in PBS. For isotype controls, beads were incubated in solutions containing equivalent concentrations of control antibodies. Antibody coupling was carried out for 2 h at room temperature with constant agitation.

After coupling, beads were washed, incubated overnight with 200 μL protein lysates (from 2 × 10⁷ cells) adjusted to 500 μL with lysis buffer (1% (v/v) NP40, 130 mM NaCl, 50 mM HEPES, Protease Inhibitor cocktail (05892791001, Merk), and washed sequentially with lysis and washing buffers (130 mM NaCl, 50 mM HEPES). Beads were subsequently resuspended in 100 μL SDS sample buffer (NuPage Bolt LDS buffer) and Sample Reducing Agent (Thermo Fisher Scientific), and incubated at 25 °C for 30 min, and the supernatant was collected for further analysis.

### Mass spectrometry analysis

Beads were resuspended in 100–200 μL of a suitable buffer for proteolytic digestion, such as 20–50 mM Tris–HCl or HEPES (pH 7.5). Two microliters of sequencing-grade trypsin (100 ng μL⁻^1^) were added, and the sample was incubated overnight (12–18 h) at 37 °C in a shaker to prevent bead settling. Subsequently, an additional 2 μL of sequencing-grade trypsin (100 ng μL⁻^1^) was added, followed by another overnight incubation at 37 °C in a shaker. To enhance digestion, 2 μL of sequencing-grade Lys-C (e.g., 50 ng μL⁻^1^, rLys-C from Promega) was added, and the sample was incubated once more overnight at 37 °C in a shaker. Beads were centrifuged, and the supernatant was collected, acidified with 2% TFA (pH < 2) and desalted using C18 ultramicro spin columns (Nest Group/Harvard Scientific Ultramicro Spin-Columns C18) following the manufacturer’s instructions. The eluate from the desalting column was dried in a speed vacuum concentrator, and the dry peptide mixture was stored at −20 °C until LC–MS/MS analysis.

Before analysis, the peptide mixture was dissolved in 3 µl 30% (v/v) formic acid and diluted with 20 µl water (final formic acid concentration 3.9%) and transferred into an HPLC vial. 5 µL were injected for LC–MS/MS analysis. The analyses were performed with a Q-Exactive HF mass spectrometer (Thermo Fisher Scientific) hyphenated to a nanoflow UPLC system (Sciex, Darmstadt, Germany). The mass spectrometer was operated in data-dependent acquisition (DDA, Top10) mode, and peptides were separated by a linear gradient from water, 0.1% (v/v) formic acid, and 60% (v/v) acetonitrile,0.1% (v/v) formic acid over 90 min.

Mass spectrometry data were analyzed with Progenesis QIP V4.2 with Mascot as the database search program. Label-free protein quantification (Mi3) was conducted to assess quantitative differences between samples, allowing differentiation of true interactors from experimental background based on protein abundance rather than mere presence or absence. Additionally, protein identifications were enriched with supplementary information, such as Gene Ontology (GO) annotations for molecular function or localization, using available commercial and academic software to support data interpretation.

A detailed description of chemicals, instrumentation and software parameters is provided in Additional File 6. The proteomic data have been deposited with the PRIDE database EMBL-EBI (https://www.ebi.ac.uk/pride/).

### Lipid analysis

Mass spectrometry-based lipid analysis was performed by Lipotype GmbH (Dresden, Germany) as described [60]. Lipids were extracted using a chloroform/methanol procedure [61]. Samples were spiked with internal lipid standard mixture containing: cardiolipin 14:0/14:0/14:0/14:0 (CL), ceramide 18:1;2/17:0 (Cer), diacylglycerol 17:0/17:0 (DAG), hexosylceramide 18:1;2/12:0 (HexCer), lyso-phosphatidate 17:0 (LPA), lyso-phosphatidylcholine 12:0 (LPC), lyso-phosphatidylethanolamine 17:1 (LPE), lyso-phosphatidylglycerol 17:1 (LPG), lyso-phosphatidylinositol 17:1 (LPI), lyso-phosphatidylserine 17:1 (LPS), phosphatidate 17:0/17:0 (PA), phosphatidylcholine 17:0/17:0 (PC), phosphatidylethanolamine 17:0/17:0 (PE), phosphatidylglycerol 17:0/17:0 (PG), phosphatidylinositol 16:0/16:0 (PI), phosphatidylserine 17:0/17:0 (PS), cholesterol ester 20:0 (CE), sphingomyelin 18:1;2/12:0;0 (SM), triacylglycerol 17:0/17:0/17:0 (TAG). After extraction, the organic phase was transferred to an infusion plate and dried in a speed vacuum concentrator. The dry extract was re-suspended in 7.5 mM ammonium formiate in chloroform/methanol/propanol (1:2:4, V:V:V). All liquid handling steps were performed using Hamilton Robotics STARlet robotic platform with the Anti-Droplet Control feature for organic solvents pipetting.

Samples were analyzed by direct infusion on a QExactive mass spectrometer (Thermo Fisher Scientific) equipped with a TriVersa NanoMate ion source (Advion Biosciences, Ithaca, NY, USA). Samples were analyzed in both positive and negative ion modes with a resolution of Rm/z = 200 = 280,000 for MS and Rm/z = 200 = 17,500 for MSMS experiments, in a single acquisition. MSMS was triggered by an inclusion list encompassing corresponding MS mass ranges scanned in 1 Da increments [62]. Both MS and MSMS data were combined to monitor CE, DAG, and TAG ions as ammonium adducts; LPC, LPC O-, PC, PC O-, as formiate adducts; and CL, LPS, PA, PE, PE O-, PG, PI, and PS as deprotonated anions. MS only was used to monitor LPA, LPE, LPE O-, LPG, and LPI as deprotonated anions; Cer, HexCer, and SM as formiate adducts.

Data were analyzed with in-house developed lipid identification software based on LipidXplorer [63, 64]. Data post-processing and normalization were performed using an in-house developed data management system. Only lipid identifications with a signal-to-noise ratio > 5, and a signal intensity fivefold higher than in corresponding blank samples were considered for data extraction.

Next, lipids were annotated to Goslin nomenclature [65] and standardized to the total lipid amount (mol%). Differential analysis was performed on contrasts of HEK293T cells without and with TMEM56 overexpression using software R 4.4.2 (R Core Team, Vienna, Austria) and packages "lipidr” [66] and “lipidSigR" [67]. Lipid set enrichment analysis (LSEA; lipidr) was carried out on ranking adjusted p-values derived from differential analysis with a minimum of 100 lipid molecules present for the respective lipid set. Significant lipid sets were identified based on p-value < 0.01 and a minimum number of 10 different lipid species. Differentially regulated individual lipid species were identified by adjusted *p*-value < 0.05.

### Statistical analyses

The experimental data were analyzed using the Mann–Whitney U test, Student's t test, and one-way analysis of variance (ANOVA) models where appropriate. Statistical differences between groups for non-parametric data were assessed using the Kruskal–Wallis test followed by Dunn’s post-hoc test with Bonferroni correction. For parametric data, post-hoc comparisons were performed using Tukey’s honest significance test or Bonferroni-corrected t-tests to identify significant differences between groups. Assumptions of normality and homogeneity of variances were checked where applicable. P-values of less than 0.05 were considered statistically significant. For lipid profile analyses, genotype effects on individual lipids were explored using linear models with empirical Bayes moderation using R/Bioconductor package limma [68]. Confidence intervals were estimated by bootstrapping. All statistical analyses were performed with GraphPad Prism (version 7; GraphPad Software, La Jolla, USA) and R (version 4.4.3; R Core Team, Vienna, Austria) using the packages FSA [69] and rstatix [70]. Results are given as mean ± standard deviation (SD). P-values are indicated as *p* ≤ 0.05, *p ≤ 0.01, **p ≤ 0.001, ***p ≤ 0.0001.

## Supplementary Information

Additional file 1.

Additional file 2.

Additional file 3.

Additional file 4.

Additional file 5.

Additional file 6.

## Data Availability

All data generated or analyzed during this study are included in this published article and its additional files.

## Abbreviations

CDS: Coding sequence
CFU-E: Erythroid colony forming unit
d/hpf: Days/hours post fertilization
GMP: Granulocyte-macrophage progenitors
HexCer: Hexosylceramide
HS(P)C: Hematopoietic stem (and progenitor) cells
kd: Knockdown
KO: Knock-out
LT-HSC: Long-term hematopoietic stem cells
MO: Morpholino oligonucleotide
oe: Overexpression
PA: Phosphatidic acid
PGC: Primordial germ cell
S1P: Sphingosine-1-phosphate
SDF-1: Stromal cell-derived factor 1
TLC: TRAM-LAG1-CLN8
wt: wildtype
