# Supplementary material for: TRAM-LAG1-CLN8 domain-containing protein TMEM56 regulates cell migration by changing intracellular ceramide levels
Source: BMC Biol. 2026 May 5;24:109. doi: 10.1186/s12915-026-02614-7 (PMC13147617; doi:10.1186/s12915-026-02614-7)
Supplement: Supplementary file 2 — Additional file 2. [file 12915_2026_2614_MOESM2_ESM.docx]

**
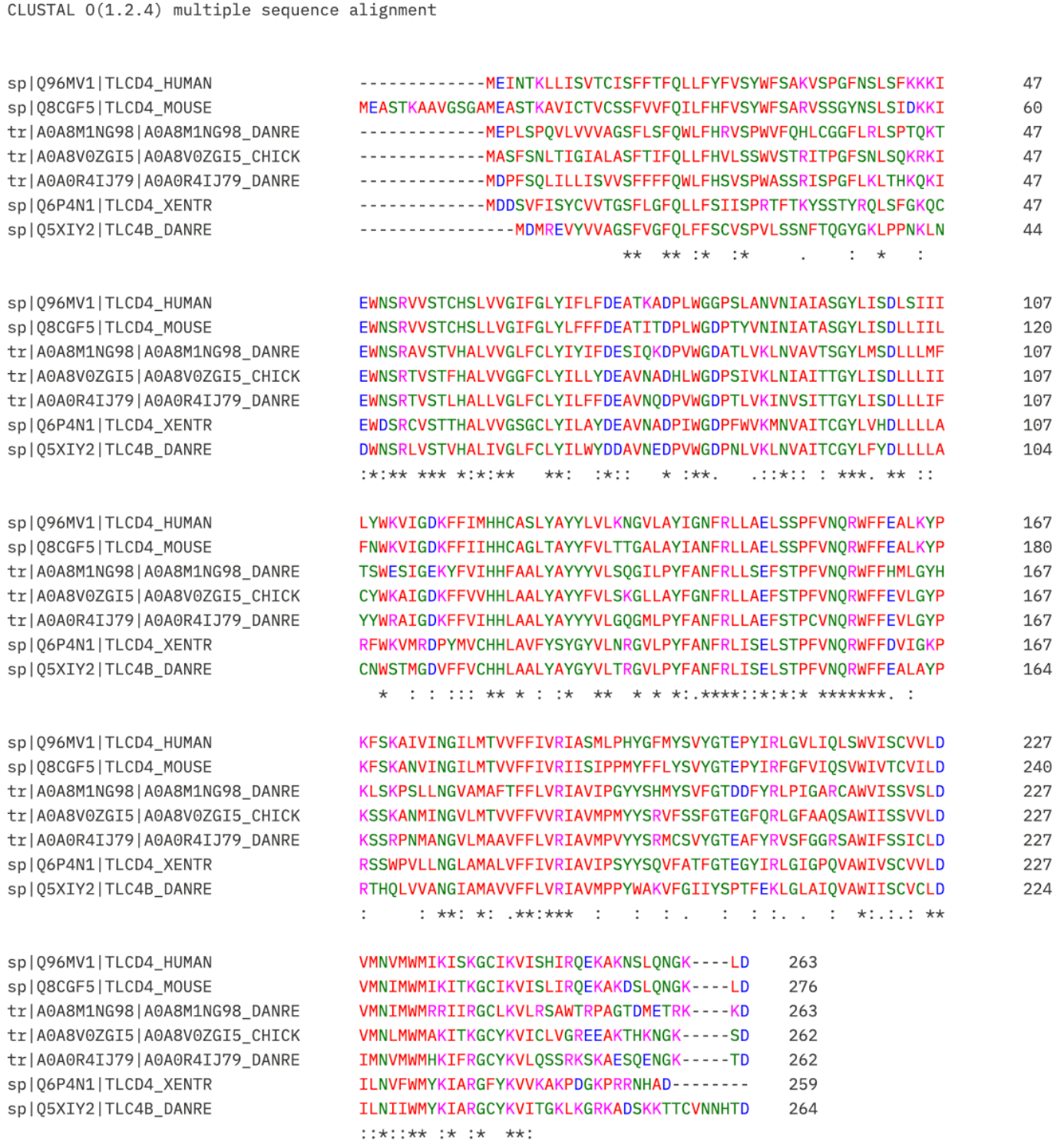
**

**Fig. S1.** Conserved homology of TMEM56. Clustal O multiple sequence alignment analysis of TMEM56 comparing protein sequence from human, mouse, chicken, Xenopus and zebrafish [1]. Colors indicate chemical properties of amino acids (red, small hydrophobic; blue, acidic; magenta, basic; green, hydroxyl; grey, unusual amino acid). Symbols below indicate the degree of conservation ('*' Exact, ':' Conserved Substitution, '.' Semi-conserved substitution). Underlined: TLC domain.

[1] Madeira F, Madhusoodanan N, Lee J, et al. The EMBL-EBI Job Dispatcher sequence analysis tools framework in 2024. Nucleic Acids Research. 2024 Jul;52(W1):W521-W525. DOI: 10.1093/nar/gkae241.


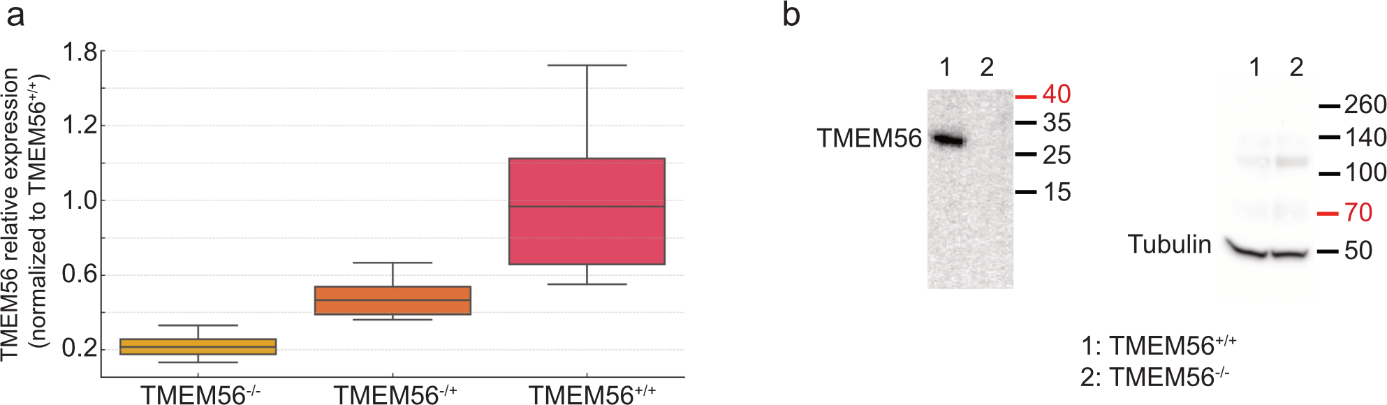


**Fig. S2.** TMEM56 expression analysis in murine liver by qPCR and Western blot. **(a)** Quantitative PCR analysis of TMEM56 expression in adult murine liver across different genotypes. Expression levels were normalized to GAPDH and are shown relative to wild-type (TMEM56+/+) animals. **(b)** Western blot analysis of murine liver tissue lysates of TMEM56^+/+^ and TMEM56^-/-^ mice. Blots were probed with anti-TMEM56 antibody (left) or anti-Tubulin antibody (right) as loading control.


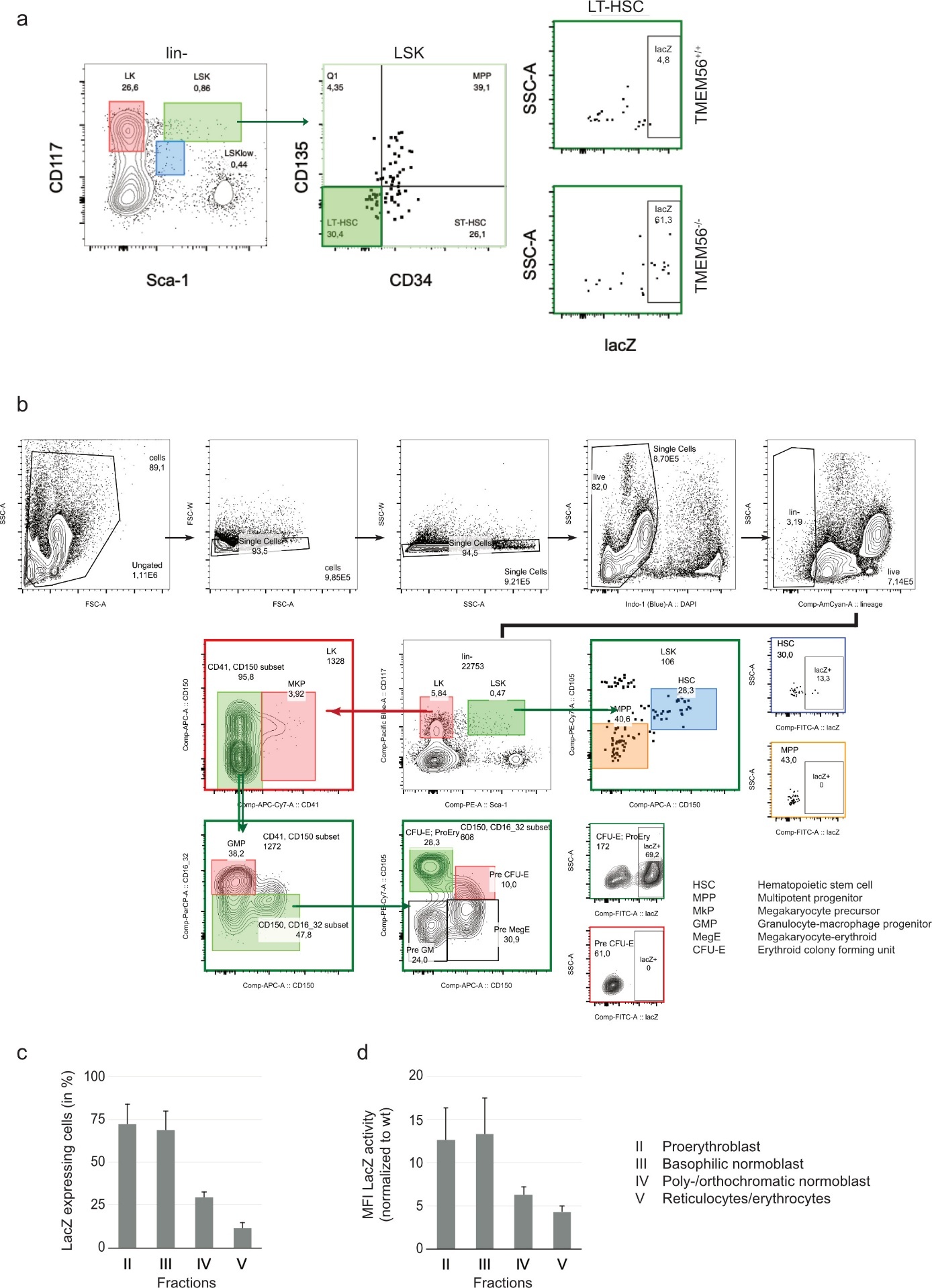


**Fig. S3.** Indirect TMEM56 expression analysis in flow cytometry. **(a)** Representative image of a TMEM56 expression analysis in hematopoietic stem cells. TMEM56^+/+^ and TMEM56^-/-^ BM cells were stained with lineage markers, Sca1, CD35, CD117, CD135, lacZ substrate and DAPI. Gating strategy includes doublets and dead cell removal. **(b)** Murine bone marrow cells were stained with antibodies against Sca1, CD16/32, CD41, CD71, CD105, CD117, CD150, Ter119 and a cocktail of mature blood cell lineage markers (Lin) as well as DAPI to exclude dead cells and lacZ substrate. **(c)** Quantitative analysis of LacZ positive cells within each fraction. Mean values and SD, n=4. (**d)** LacZ activity in each fraction depicted as normalized median fluorescence intensity (MFI). Mean values and SD, n=4.
