# Supplementary material for: TRAM-LAG1-CLN8 domain-containing protein TMEM56 regulates cell migration by changing intracellular ceramide levels
Source: BMC Biol. 2026 May 5;24:109. doi: 10.1186/s12915-026-02614-7 (PMC13147617; doi:10.1186/s12915-026-02614-7)
Supplement: Supplementary file 3 — Additional file 3. [file 12915_2026_2614_MOESM3_ESM.docx]

**Supplementary Information on Zebrafish Experiments**

To assess SDF-1/CXCR4-dependent migration of primordial germ cells (PGCs) during embryonic development *in vivo* using antisense morpholino oligonucleotides (MOs), three translation-blocking MOs targeted the zebrafish paralogs *tlcd4b* (formerly annotated as *tmem56/tmem56b*), *tlcd4a* (formerly *tmem56a*), and *si:dkey-10f21.4* (formerly annotated as *transmembrane protein 56-B-like isoform X1*). Injection of single or pairwise morpholino combinations did not induce changes in PGC migration, suggesting that functional redundancy between the three paralogs masked the phenotype in single or double knockdowns. Therefore, all three were co-injected as a pooled mix to ensure comprehensive knockdown of TMEM56-related function in zebrafish.

To exclude unspecific or toxic effects, we first performed a dose–response analysis of pooled TMEM56 MOs. Embryo survival remained high across all concentrations tested, with no evidence of general toxicity relative to control MOs. Only at the highest cumulative dose (60 ng) did we observe a slight decrease in survival, indicating mild sensitivity at elevated morpholino levels (Fig. S4). Overall, these results demonstrate that TMEM56-targeting morpholinos are well tolerated up to 45 ng and that the phenotypes described below cannot be attributed to general toxicity.

At tolerated doses, injection of pooled TMEM56-specific antisense MOs into 1-cell stage zebrafish embryos resulted in severe developmental abnormalities at 24 hours post fertilization (hpf), including defects in body axis elongation and head formation. The observed phenotypes are consistent with impaired cell migration during gastrulation and somitogenesis (Fig. 1c of the main manuscript). Despite these overall morphological abnormalities, PGC migration is highly robust to alterations in embryo shape, which allows assessment of normal and ectopic PGC migration in morphant embryos [1]. Indeed, we observed a substantial proportion of PGCs failing to reach their bilateral destinations near the future gonads, in contrast to control MO-injected embryos (Fig. S5a). Also, TMEM56 knockdown led to a significant reduction in the number of PGCs at 24 hpf (Fig. S5b). Importantly, co-injection of synthetic *tmem56* (now annotated as *tlcd4b*) mRNA, modified to be resistant to MO binding, but not eGFP control mRNA rescued the morphological defects, PGC number, and migration phenotype (Fig. 1c, Fig. S5a-b), confirming the specificity of the knockdown.

1. Weidinger G, Wolke U, Köprunner M, Klinger M, Raz E. Identification of tissues and patterning events required for distinct steps in early migration of zebrafish primordial germ cells. Development. 1999;126:5295–307. doi:10.1242/dev.126.23.5295.


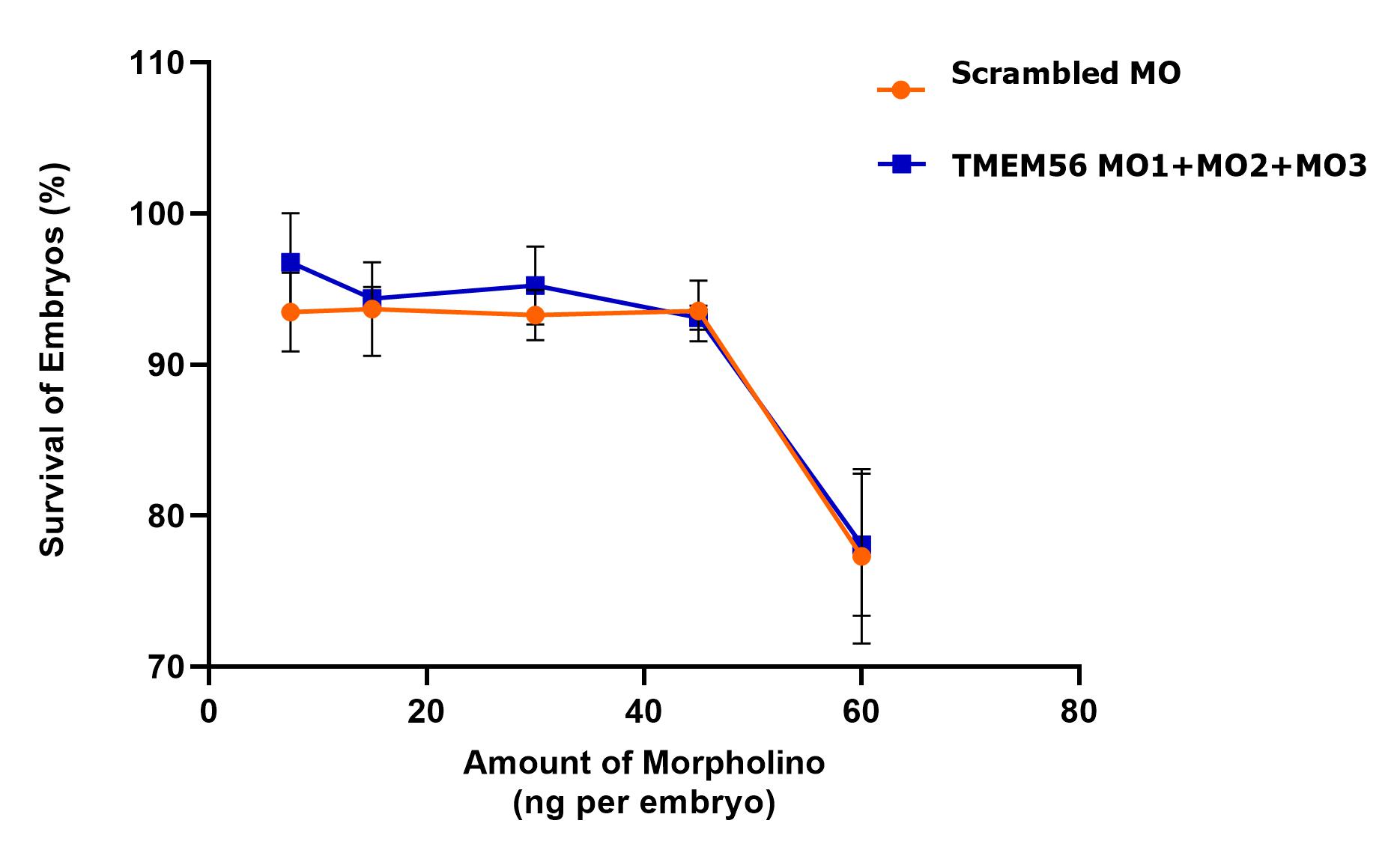


**Fig. S4.** TMEM56 morpholino dose response. The relationship between increasing morpholino concentrations (7.5–60 ng per embryo) and zebrafish embryo survival for the TMEM56-targeting morpholino (TMEM56 MO1+MO2+MO3) and the scrambled control groups. Each point represents the mean percentage of surviving embryos from three independent experiments, with error bars indicating the standard deviation (SD). At the highest tested dose (60 ng), survival declined in both groups, indicating a potential onset of dose-related sensitivity or nonspecific effects at elevated morpholino concentrations. Statistical analysis using the Mann–Whitney U test showed no significant differences between groups at any dose (p > 0.05).

**
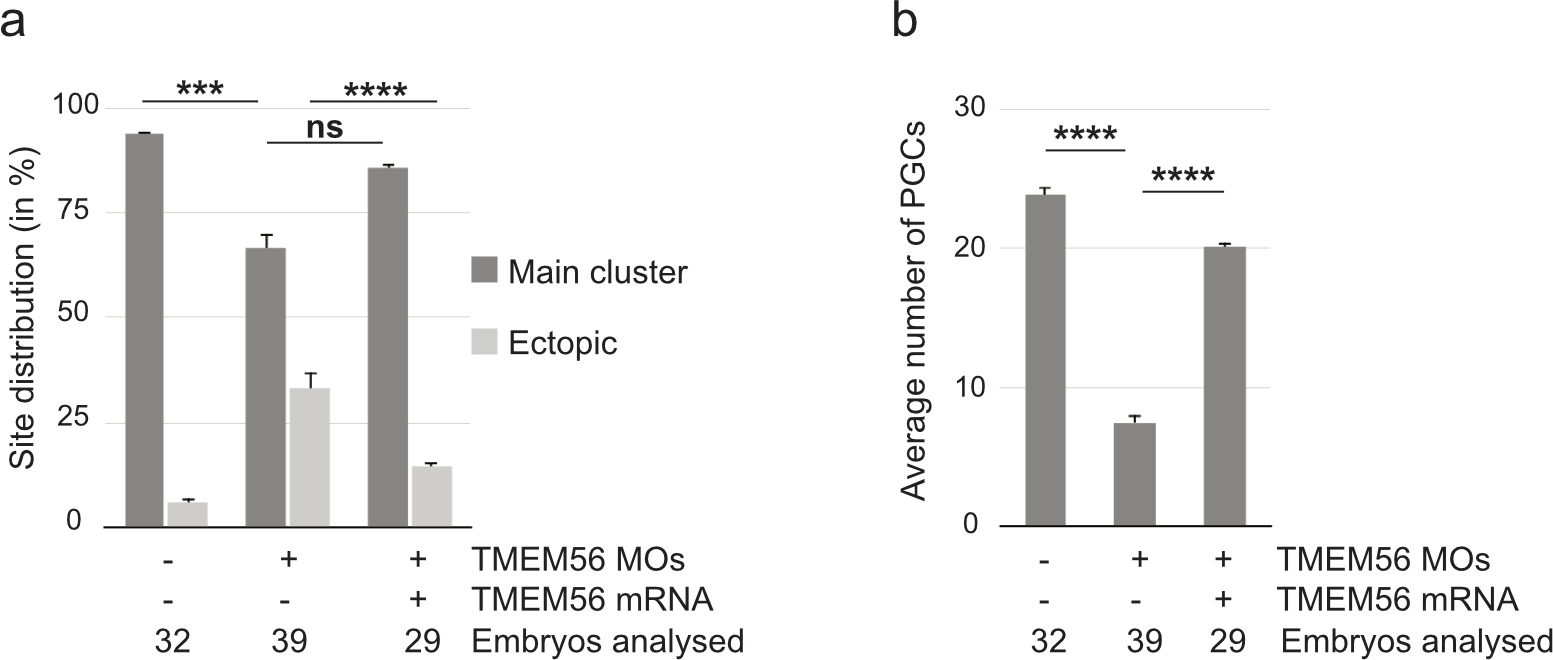
**

**Fig. S5.** Quantitative analysis of primordial germ cell (PGC) migration. **(a)** The distribution of PGCs (localized in the main cluster or ectopic) is represented as means of percentage ± standard deviation (SD). Statistical differences between groups were assessed using the Kruskal-Wallis test followed by Dunn’s post-hoc test with Bonferroni correction. **(b)** Numbers of PGCs are shown as mean ± SD. Statistical significance between groups was assessed by the Kruskal-Wallis test followed by Dunn’s post-hoc test with Bonferroni correction.
