# Supplementary material for: TRAM-LAG1-CLN8 domain-containing protein TMEM56 regulates cell migration by changing intracellular ceramide levels
Source: BMC Biol. 2026 May 5;24:109. doi: 10.1186/s12915-026-02614-7 (PMC13147617; doi:10.1186/s12915-026-02614-7)
Supplement: Supplementary file 6 — Additional file 6. [file 12915_2026_2614_MOESM6_ESM.docx]

# Material & Methods Supplement

## Materials

### Abreviations

| NH_4_HCO_3_ | Ammoniumbicarbonate |
| --- | --- |
| BSA | Bovine Serum Albumin |
| CBB | Coomassie Brilliant Blue |
| DTT | Dithiothreitol |
| FA | Formic Acid |
| IAA | Iodacetamide |
| MWCO | Molecular Weigth Cut-Off |
| RIPA | RadioImmunoPrecipitation Assay |
| SDS | Sodium Dodecylsulfate |
| SDT | Sodium Dodecylsulfate - Dithiothreitol |
| TEAB | Triethylammoniumhydrogencarbonate *(from triethylammoniumbicarbonate)* |

### Chemicals

| Item (Chemicals) | Manufacturer (Supplier) | Order Number |
| --- | --- | --- |
|  |  |  |
| BSA (>99%) | Thermo Pierce (Part of BCA-Kit or separate) |  |
| DTT | Sigma | D-5545 |
| NH_4_HCO_3_ (Ammoniumbicarbonate) | Sigma | A-6141 |
| IAA | Sigma | I-1149 |
|  |  |  |
| Water HPLC grade | Merck, Darmstadt, Germany | 1.15333.2500 |
| Acetonitrile HPLC Grade | Merck, Darmstadt, Germany | 1.00029.2500 1.00030.2500 |
| Formic Acid p.a. | Merck, Darmstadt, Germany | 1.00264.0100 |
| Trypsin Gold sequencing grade | Promega, Walldorf, Germany | V5280 |
| rLys-C | Promega, Walldorf, Germany | V1671 |
|  |  |  |

### General Materials

| Item (Chemicals) | Manufacturer (Supplier) | Order Number |
| --- | --- | --- |
| 0.65ml Reaction Tubes | Sorensen  (Supplier C. Roth, KA, Germany) | A- |
| 1.5ml/2.0ml Reaction Tube  (no coating or modifications) | Eppendorf, Hamburg, Germany |  |
|  |  |  |

### Instrumentation

#### UPLC-System Eksigent LC425

| Instrument / Material | Manufacturer (Supplier) | Comments |
| --- | --- | --- |
| Eksigent 425 UPLC System | Sciex, Darmstadt, Germany | Nanoflow UPLC System |
| Acclaim PepMap 100 C18, 2 µm, 75 µm x 2 cm, Acclaim PepMap C18, 2 µm, 75 µm x 15 cm | ThermoScientific, Idstein, Germany | Vented-Column Setup #164535 #164534 |
| Picotip Needle 20 µm / 10 µm | NewObjectives, Woburn, USA |  |

#### Mass Spectrometer Q-Exactive HF -DDA-

| Instrument / Parameter | Value | Comments |
| --- | --- | --- |
| Q-Exactive HF |  | DDA-Mode |
| MS1 |  |  |
| Polarity | positive |  |
| Resolution | R120000 at m/z 200 |  |
| AGC | 3x 10E6 |  |
| Max. Fill Time | 100 ms |  |
| Lock Mass | m/z 445.120025 | Dodecamethylcyclohexasiloxane [1] |
| Range | m/z 395-1500 |  |
| Inclusion | 5.0 ppm | Peptide Retention Time Standard |
| Dynamic Exclusion | 3.0 ppm |  |
| Spectrum Data Type | Profile |  |
| Picotip Needle | 20 µm / 10 µm |  |
| Voltage | 2.3-2.7kV | experiment dependent adjustment |
|  |  |  |
| MS2 | Top10 | HCD |
| Microscans | 1 |  |
| Resolution | R15000 at m/z 200 |  |
| AGC | 1E5 |  |
| Max. Fill Time | 50 ms |  |
| Loop Count | 10 |  |
| MSX count | 1 |  |
| TopN | 10 |  |
| Isolation window | 2.0 m/z |  |
| Isolation Offset | 0.3 m/z |  |
| Scan Range | 200 to 2000 m/z |  |
| Fixed 1^st^ Mass | --- |  |
| Norm. Collision Energy | 27 | (NCE) |
| Spectrum Data Type | Centroid |  |
| Minimum AGC Target | 4.00E3 |  |
| Intensity Threshold | 8.0E4 |  |
| Apex Trigger | --- |  |
| Charge states | Unassigned, 1, 6-8,>8 | (rejected) |
| Dynamic Exclusion | 20s, 3ppm | If idle…. pick others |

### Software

| Instrument / Material | Manufacturer (Supplier) | Comments |
| --- | --- | --- |
| MSConvert V3.0 [2,3] | Proteowizard, CDN | File Conversion Tool |
| MASCOT V2.6 [4] | MatrixScience, London, UK | Protein Identification Software matrixscience.com |
| Progenesis QIP V4.2 | Nonlinear Dynamics (Waters), Newcastle upon Tyne, UK | Quantitative Proteomics Software nonlinear.com |

### Mascot Parameters

| **Mascot Parameter QE-HF** | Value | Comments |
| --- | --- | --- |
| **Version** | 2.6 |  |
| MS Tolerance | 10 ppm |  |
| Protease | Trypsin |  |
| Missed Cleavages | 3 |  |
| Fixed Modifications | none | On-bead digest |
| Variable Modifications | Acetyl- N-Protein, Oxidation (M) |  |
| MS/MS Tolerance | 30 mmu |  |
| Instrument | ESI-Quad |  |
| Databases | Uniprot Human Reference Proteome  In addition in-house databases for: Contaminants, Enzymes Standards and Tags |  |
| Decoy | Yes |  |

## References

[1] A. Schlosser, R. Volkmer-Engert, Volatile polydimethylcyclosiloxanes in the ambient laboratory air identified as source of extreme background signals in nanoelectrospray mass spectrometry, Journal of Mass Spectrometry 38 (2003) 523–525. https://doi.org/10.1002/jms.465.

[2] M.C. Chambers, B. MacLean, R. Burke, D. Amodei, D.L. Ruderman, S. Neumann, L. Gatto, B. Fischer, B. Pratt, J. Egertson, K. Hoff, D. Kessner, N. Tasman, N. Shulman, B. Frewen, T.A. Baker, M.Y. Brusniak, C. Paulse, D. Creasy, L. Flashner, K. Kani, C. Moulding, S.L. Seymour, L.M. Nuwaysir, B. Lefebvre, F. Kuhlmann, J. Roark, P. Rainer, S. Detlev, T. Hemenway, A. Huhmer, J. Langridge, B. Connolly, T. Chadick, K. Holly, J. Eckels, E.W. Deutsch, R.L. Moritz, J.E. Katz, D.B. Agus, M. MacCoss, D.L. Tabb, P. Mallick, A cross-platform toolkit for mass spectrometry and proteomics, Nature Biotechnology 30 (2012) 918–920. https://doi.org/10.1038/nbt.2377.

[3] D. Kessner, M. Chambers, R. Burke, D. Agus, P. Mallick, ProteoWizard: Open source software for rapid proteomics tools development, Bioinformatics 24 (2008) 2534–2536. https://doi.org/10.1093/bioinformatics/btn323.

[4] D.N. Perkins, D.J.C. Pappin, D.M. Creasy, J.S. Cottrell, Probability-based protein identification by searching sequence databases using mass spectrometry data, Electrophoresis 20 (1999) 3551–3567. https://doi.org/10.1002/(SICI)1522-2683(19991201)20:18<3551::AID-ELPS3551>3.0.CO;2-2.
